# Immunocytochemical Localization of XRCC1 and γH2AX Foci Induced by Tightly Focused Femtosecond Laser Radiation in Cultured Human Cells

**DOI:** 10.3390/molecules26134027

**Published:** 2021-07-01

**Authors:** Alexandr Zalessky, Yuriy Fedotov, Elizaveta Yashkina, Viktor Nadtochenko, Andreyan N. Osipov

**Affiliations:** 1 N.N. Semenov Federal Research Center for Chemical Physics, Russian Academy of Sciences, 119991 Moscow, Russia; aleksandr.zalesskij@phystech.edu (A.Z.); ufedotov456@gmail.com (Y.F.); yashkinaliz@gmail.com (E.Y.); nadtochenko@gmail.com (V.N.); 2 Moscow Institute of Physics and Technology, Dolgoprudny, 141700 Moscow, Russia; 3 State Research Center—Burnasyan Federal Medical Biophysical Center of Federal Medical Biological Agency (SRC—FMBC), 123098 Moscow, Russia; 4 Chemistry Department, Lomonosov Moscow State University, 119991 Moscow, Russia

**Keywords:** femtosecond laser radiation, DNA double-strand breaks, XRCC1, γH2AX, human cells, A549

## Abstract

To assess the prospects for using intense femtosecond laser radiation in biomedicine, it is necessary to understand the mechanisms of its action on biological macromolecules, especially on the informational macromolecule—DNA. The aim of this work was to study the immunocytochemical localization of DNA repair protein foci (XRCC1 and γH2AX) induced by tightly focused femtosecond laser radiation in human cancer A549 cells. The results showed that no XRCC1 or γH2AX foci tracks were observed 30 min after cell irradiation with femtosecond pulses of 10^11^ W∙cm^−2^ peak power density. An increase in the pulse power density to 2 × 10^11^ W∙cm^−2^ led to the formation of linear tracks consisting both of XRCC1 and γH2AX protein foci localized in the places where the laser beam passed through the cell nuclei. A further increase in the pulse power density to 4 × 10^11^ W∙cm^−2^ led to the appearance of nuclei with total immunocytochemical staining for XRCC1 and γH2AX on the path of the laser beam. Thus, femtosecond laser radiation can be considered as a tool for local ionization of biological material, and this ionization will lead to similar effects obtained using ionizing radiation.

## 1. Introduction

Femtosecond laser radiation in the near infrared range (800–1100 nm) is widely used in biological research, including as an ultra-precise scalpel for nanosurgical treatment [1,2]. The physicochemical basis of this application is based on the principles of nonlinear absorption of laser pulses with a high peak power and the subsequent formation of low-density plasma in the absorption region of a femtosecond laser pulse [3]. Exposure of biological material to this plasma can lead to the desired radiobiological effects. Using the principles of nonlinear absorption of laser radiation, it becomes possible to concentrate the effect of laser radiation on cells lying at a macroscopic distance from the surface.

To assess the prospects for using femtosecond laser radiation in biomedicine, it is necessary to understand the mechanisms of its action on biological macromolecules, especially on the informational macromolecule—DNA. Of particular interest is the formation of crucial DNA damage-double-strand breaks (DSBs). DSB repair occurs slowly and often (up to 80%) incorrectly, with frequent formation of various genetic disorders, while the impossibility of DSBs repair leads to cell death [4,5,6]. One of the most sensitive and informative methods for studying the formation and repair of DSBs is the immunocytochemical analysis of microdynamic structures consisting of hundreds, even thousands, of copies of proteins involved or associated with DNA repair processes (protein repair foci). The most commonly used DSB repair marker is phosphorylated core histone H2AX (γH2AX) foci. Phosphorylation of H2AX is carried out by phosphatidylinositol-3-kinases ATM, ATR, and DNA-PKcs in response to DSB formation [7,8]. DSBs can be formed both directly and during the formation of two or more single-strand breaks (SSBs) when the phosphate backbones of the two complementary DNA strands are broken simultaneously via free-radical attacks [9]. Therefore, in order to understand the nature of DSBs generated by femtosecond laser radiation, it is extremely important to investigate oxidative DNA damage like SSBs. One of the major coordinating proteins for the repair of such DNA damage by excision repair mechanisms is X-ray repair cross-complementing protein 1 (XRCC1) [10].

The aim of this work was to study the immunocytochemical localization of XRCC1 and γH2AX foci induced by femtosecond laser radiation in human cancer A549 cells (lung adenocarcinoma).

The common peak femtosecond laser intensities in surgical treatment are in the range of 1 to 50 × 10^12^ W∙cm^−2^ (with pulse duration of 100–300 fs, N.A. of the objective 0.7–1.4, and repetition rate 1–80 MHz or 1 kHz) [3,11,12]. In the present study we used intensities below this common region and even more, below the threshold of vapor-gas bubble formation in the cell cytoplasm (6 × 10^11^ W∙cm^−2^), which was determined in our previous work [13]. An example of developing a near-infrared region femtosecond laser system for DNA damage induction and repair studies has been already reported [14,15]. In the present study we concentrate on several main points: firstly, to reveal the dependence of the DNA damage effects on the femtosecond pulse peak intensity (in the region below commonly used in femtosecond laser surgical treatments), and secondly, to investigate XRCC1 and γH2AX foci colocalization after exposure to different pulse energies.

## 2. Results

To irradiate the samples, we used trains of femtosecond pulses with a carrier wavelength of 794 nm; energies of 0.5, 1, and 2 nJ; a duration of 100 fs; and a repetition rate of 80 MHz. The diameter of the laser beam waist was 2.5 μm, and the power density for the selected pulse energies was 1, 2, and 4 × 10^11^ W∙cm^−2^, respectively.

**Figure 1:**
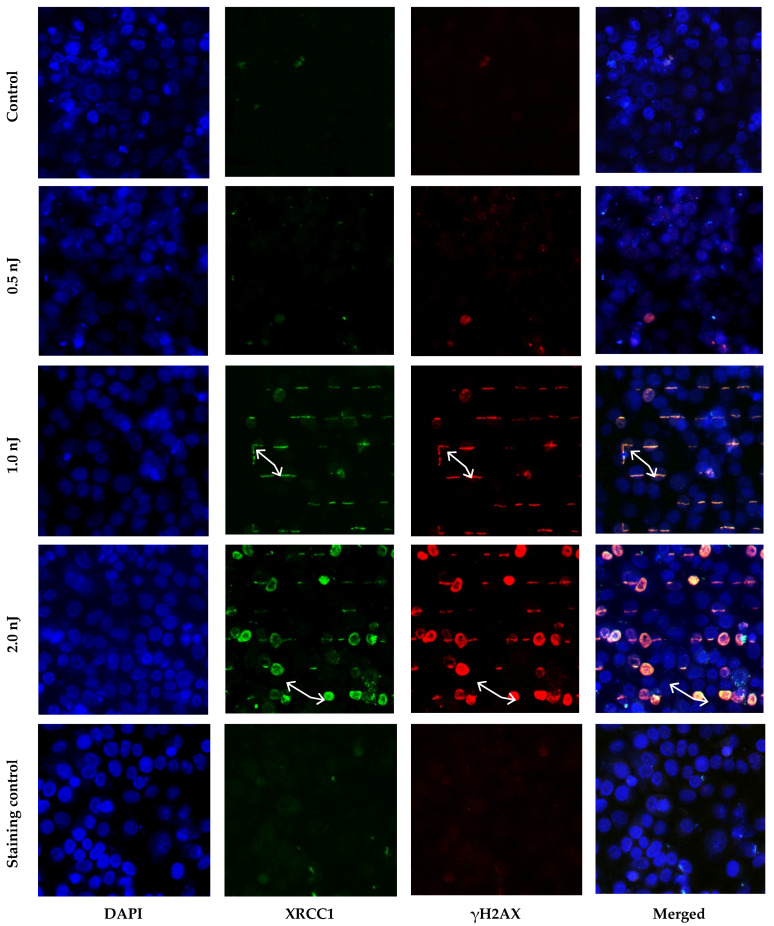
Microscopy image of immunocytochemically stained cell nuclei 30 min after exposure to femtosecond laser radiation with a pulse energy of 0.5 nJ (power density 1 × 10^11^ W∙cm^−2^), 1.0 nJ (power density 2 × 10^11^ W∙cm^−2^), and 2.0 nJ (power density 4 × 10^11^ W∙cm^−2^). Arrows show foci tracks (1.0 nJ) or pan-stained nuclei (2.0 nJ). Staining control cells irradiated with a pulse energy of 2.0 nJ and stained with only secondary antibodies.

The results of immunocytochemical analysis of the XRCC1 and γH2AX foci showed that no linear foci tracks for either protein were observed 30 min after irradiation of cells with femtosecond pulses with an energy of 0.5 nJ. An increase in the pulse energy to 1.0 nJ led to the formation of linear tracks consisting of XRCC1 and γH2AX protein foci localized in the places where the laser beam passes through the cell nuclei (Figure 1). 

Visually, the tracks were very similar to the tracks formed after the irradiation of cells with ionizing radiation with a high linear energy transfer. It can be assumed that femtosecond laser radiation can be used to simulate the processes of DNA damage by high-LET ionizing radiation in living cells. The colocalization of XRCC1 and γH2AX foci tracks indicates that femtosecond laser radiation induced simultaneously different types of DNA damage involving different repair pathways. A further increase in the pulse energy to 2.0 nJ led to the appearance of nuclei with total immunocytochemical staining for γH2AX on the path of the laser beam passage (Figure 1). Such staining of γH2AX nuclei may indicate the onset of cell death by the apoptosis mechanism [16,17]. However, for a final conclusion, further studies are needed with more specific markers of apoptosis.

## 3. Discussion

**Figure 2:**
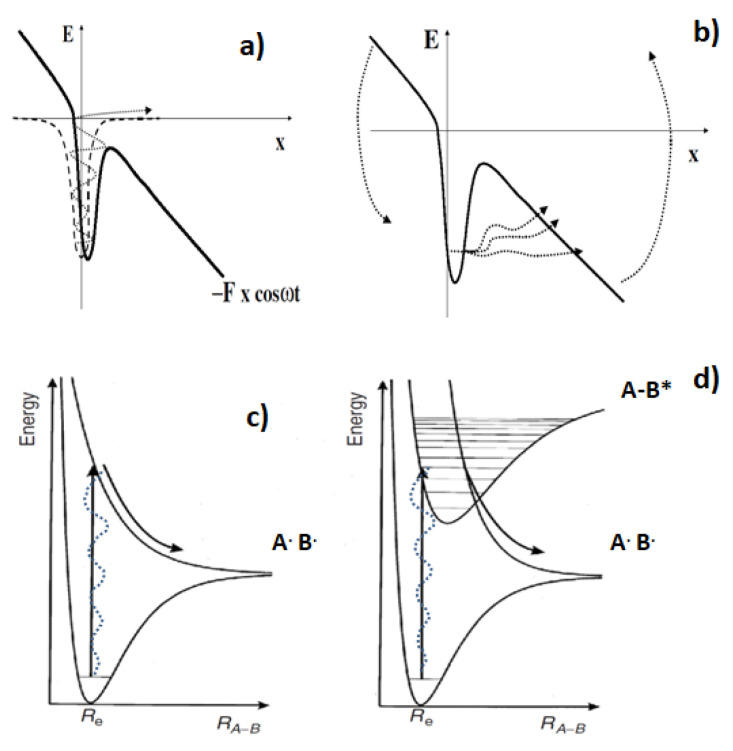
The absorption of several (n) photons can result in multiphoton dissociation (MPD), as well as multiphoton ionization (MPI). (**a**) MPI ionization, Keldysh parameter γ >> 1; (**b**) ionization according to the tunneling mechanism, γ << 1; (**c**) MPD according to direct dissociation; (**d**) MDP according to predissociation.

Using irradiance well below the optical breakdown threshold, it is possible to achieve highly localized modification of biological material [3]. The mechanisms underlying femtosecond laser nanosurgery of cells and biological tissues could be explained by chemical, thermal, and thermomechanical effects arising from low-density plasma formation under femtosecond irradiance [3,18,19]. It has been shown experimentally that the optical breakdown threshold in water is very similar to that in biological media [3,19]. The process of plasma formation mostly consists of quasi-free electron formation through the interplay of photoionization and avalanche ionization [20,21,22,23,24]. Figure 2 demonstrates that the absorption of several (n) photons can result in multiphoton dissociation (MPD), as well as multiphoton ionization (MPI). The probability of an MPI or MPD event depends on the power density of the exciting laser pulse (I). The adiabatic ionization energy of bulk liquid water is equal to *V*_0_ = 10.12 eV [25]. The ratio of the water ionization energy to the photon energy of the laser radiation *k* = *V*_0_/hν equals 6.3 (λ = 780 nm, hν = 1.6 eV). The threshold energies required for DNA ionization in aqueous solution lie in the range of 4 to 7 eV, which corresponds to the ratio *k* = *V*_0_/hν = 2.5–4.4 [26]. Keldysh showed that both multiphoton and tunnelling regimes could be described within the same framework. The Keldysh parameter γ was suggested to predict whether ionization occurs by MPI (γ >> 1) or by tunnel ionization via the following equation: $\gamma =\sqrt{\frac{V_{0}}{I\;\times 1.87\times 10^{-19}\times {\;\lambda }^{2}}}$, where *V*_0_ is in eV, I is in W/cm^2^, and λ is in nm. With a pulse energy of 1 nJ, the gamma parameter is 11.2 for water and the gamma is in the range of 7–9.5 for DNA. When the Keldysh parameter is larger (smaller) than about 1.5, photoionization is a multiphoton (tunnelling) process [27,28]. The Keldysh parameter γ >> 1 suggests that MPI dominates the initial interaction process of the laser pulse with water as well as with DNA. In the multiphoton ionization regime, the rate is P(I) = σ_n_I^n^, where σ_n_ is the multiphoton absorption coefficient for absorption of n photons with the smallest n satisfying ≥*k*·hν. A laser pulse of 100 fs duration and I ~ 10^12^ W∙cm^−2^ multiphoton ionization produces a substantial amount of free electrons with only a small collisional avalanche required to achieve critical density [24,29,30]. Critical density (=10^21^ cm^−3^) is not produced.

Chemical effects arising from MPI or MDP formation could be divided into two groups: (1) changes in the organic molecules due to reactive oxygen species (ROS) caused by water molecule modification, and (2) changes in the organic molecules due to resonant electron-molecule scattering.

The second one is of great importance from the DNA damage point of view. Biomolecule fragmentation can be initiated by the capture of electrons into an antibonding molecular orbital [31].

**Figure 3:**
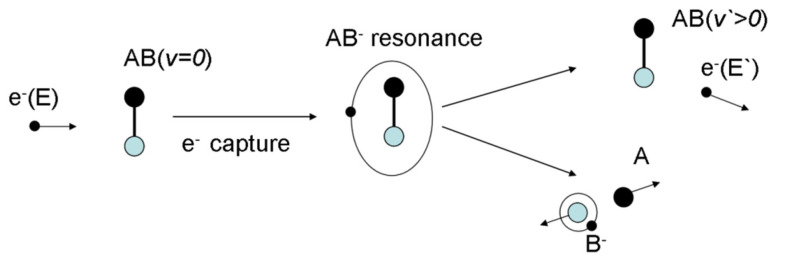
Diagram of dissociative electron attachment in a resonant electron molecule.

For an AB molecule, this process corresponds to e^−^ + AB → AB*^−^, where AB*^−^ has a repulsive potential along the A-B bound. After a time of 10^−15^ to 10^−11^ s, the dissociation along one or several specific bonds, such as AB*^−^ → A● + B^−^, could occur (Figure 3).

The resonant formation of DNA strand breaking induced by low-energy electrons is well described [32,33]. Accumulative effects of this kind can lead to the dissociation of biological structures that were exposed to low-density plasma, generated by femtosecond laser pulses.

Nonlinear absorption of laser radiation occurs in a medium during the passage of a powerful femtosecond laser pulse if its intensity is so high that it deforms the electron shells of the medium molecules and changes their optical properties [34]. As a result of such changes, the initially optically transparent medium (in a given wavelength range) begins to absorb the energy of laser radiation. Thus, by controlling the parameters of the laser radiation (such as the geometry of the laser beam, the power of the laser pulse, etc.), it becomes possible to control the area of the greatest effect of the laser radiation. This controlled absorption of laser energy leads to the formation of free electrons and, as a result, localized low-density plasma. This type of plasma allows ionization to be obtained in the region of maximum absorption of laser radiation, while minimizing thermo-mechanical effects such as shock waves and cavitation.

Therefore, femtosecond laser radiation can be considered as a tool for local ionization of biological material, and this ionization will lead to similar effects obtained using ionizing radiation.

With femtosecond lasers getting more affordable, they become a common instrument for biomedical research and applications such as multiphoton microscopy, FLIM [35], nano- and microsurgery [36,37], and so on. For this reason, data on interaction between femtosecond laser radiation and biomaterial (cells, intracellular structures, tissues) are of great importance. It has been shown in this work that femtosecond laser pulses with relatively small peak intensity (below the numbers that are commonly used in biomedical applications) could act as a highly localized ionizing tool. This effect was employed for DNA damage and repair study. We suppose that this local ionizing effect should be taken into consideration when femtosecond laser radiation is used for biomedical applications.

In general, our research showed that femtosecond laser radiation induces complex DNA damage involving different repair pathways. In our future research, the next perspective open issues of femtosecond laser radiation biological action will be solved using a higher number of cells of various lineages: (1) types and complexity of DNA lesions, (2) DNA repair efficiency of femtosecond laser-induced DNA damage, (3) death mechanisms of irradiated cells (apoptosis, necrosis, etc.), and (4) possible bystander effects in non-irradiated cells.

## 4. Materials and Methods

### 4.1. Cell Culture and Culture Conditions

The studies were carried out on a culture of human cancer cell A549 (lung adenocarcinoma) obtained from ATCC (Manassas, VA, USA). The cells were cultured in DMEM/F12 medium (Thermo Fisher Scientific, Waltham, MA, USA) containing 10% fetal bovine serum (FBS, Thermo Fisher Scientific, Waltham, MA, USA) and antibiotics (PenStrep, Paneco, Moscow, Russia) under the standard conditions of a CO_2_ incubator (37 °C, 5% CO_2_) with a change of medium once every three days. When 75–80% of the monolayer was reached, the cell culture was removed from the plastic by enzymatic means. Prior to experiments, cells were passaged into sterile 8-well glass bottom slide vials (SPL Lifesciences, Gyeonggi-do, Korea).

### 4.2. Irradiation of Cells

**Figure 4:**
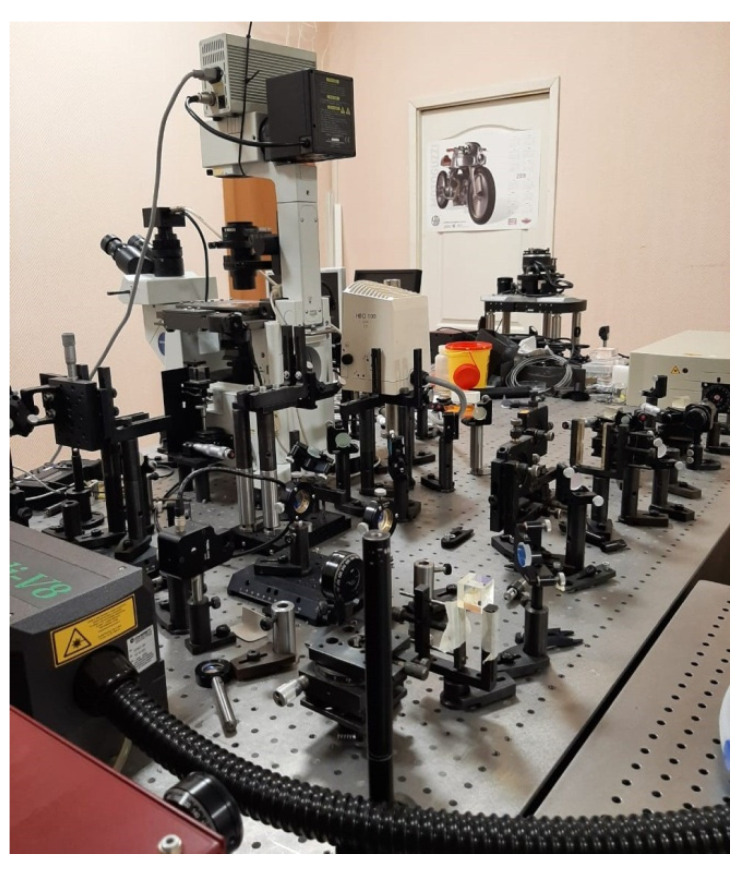
The irradiation setup.

Femtosecond laser pulses (repetition rate 80 MHz, energy up to 20 nJ) were generated by a tunable titanium-sapphire laser (Mai-Tai, Spectra-Physics, Santa Clara, CA, USA). The average power in front of the microscope objective did not exceed 400 mW and was controlled using a polarizer and a half-wave plate. Femtosecond radiation with wavelength of λ = 780 nm was coupled with an inverted microscope (Olympus IX71) using a mirror (10B20UF.25, Newport, Irvine, CA, USA) set at an angle of 450, and then focused by a microscope objective (LUCPlanFLN 60 × 0.70 NA, Olympus, Tokyo, Japan) into the object plane on the object of study (cells). The laser beam filled the entrance pupil of the objective completely. The waist diameter of the laser beam was 2·w_0_ = 1.22·λ/NA ~ 1.36 µm, Rayleigh parameter (waist length) Z_0_ = (2·π/λ)·w_0_^2^ ~ 1.65 µm. The duration of femtosecond pulses was measured in the object plane of the microscope using an autocorrelator (Avesta AA-M). The pulse time duration was 100 fs. A prism compressor was used to compensate for the group velocity dispersion in the optical elements of the setup. A motorized microscope stage was used to automate the process of laser action on cells. Image fixation and observation of the affected objects was carried out using a XIMEA xiQ MQ013MG-ON or XIMEA xiD MD061CU-SY camera mounted on a microscope. The irradiation setup picture is presented in Figure 4.

**Figure 5:**
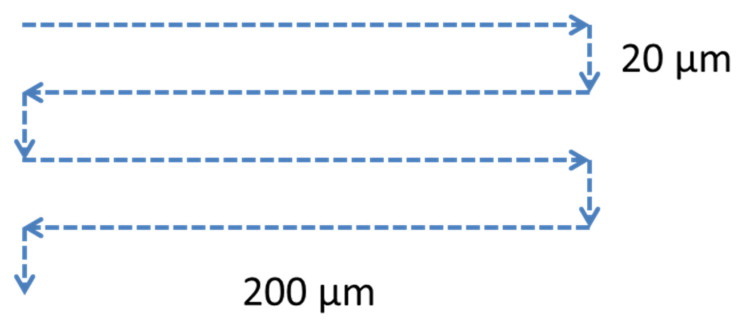
Pattern of the laser beam movement during the irradiation of the cells.

Cells were irradiated through the glass bottom of a slide vial. The bottom thickness was 1 mm. To automate the irradiation process, the motorized stage of the microscope was programmed to perform the movement pattern shown in Figure 5. The average speed of movement of the microscope stage was 13.8 µm/s, which corresponds to 5.8 × 10^6^ femtosecond pulses per 1 µm of the sample.

### 4.3. Immunocytochemical Analysis of DNA Double-Strand Break Localization

The cells were fixed with paraformaldehyde (4% in phosphate buffered saline, pH 7.4) for 20 min at room temperature 30 min after irradiation. Then, they were washed out twice with phosphate buffered saline (pH 7.4). They were permeabilized with 0.3% Triton-X100 in phosphate-buffered saline (pH 7.4) containing 2% bovine serum albumin to block nonspecific binding. The slides were incubated with primary antibodies (mouse monoclonal anti-XRCC1 antibody (clone 33-2-5, Abcam, Waltham, MA, USA) and rabbit monoclonal anti-γH2AX antibody (clone EP854(2)Y, Merck-Millipore, Burlington, VT, USA) at a dilution of 1/200) in phosphate-buffered saline (pH 7.4) containing 1% bovine serum albumin for 1 h at room temperature. Then the slides were washed out with phosphate-buffered saline (pH 7.4) and incubated at room temperature for 1 h with secondary goat anti-mouse and goat anti-rabbit IgG (H+L) antibodies conjugated with Alexa Fluor 488 and Alexa Fluor 555 fluorochromes (Life Technologies, Carlsbad, SA, USA), accordingly. Both antibodies were diluted 1/800 in phosphate-saline buffer (pH 7.4) containing 1% bovine serum albumin. DAPI containing ProLong Gold (Life Technologies, Carlsbad, SA, USA) was used to stain DNA and prevent photobleaching.

Imaging, documenting, and processing the immunocytochemical microimages was carried out on a Nikon Eclipse Ni-U fluorescent microscope (Nikon, Tokyo, Japan) equipped with a ProgRes MFcool high-resolution video camera (Jenoptik AG, Jena, Germany) using UV-2E/C filter sets (340–380 nm excitation and 435–485 nm emission), B-2E/C (465–495 nm excitation and 515–555 nm emission), and Y-2E/C (540–580 nm excitation and 600–660 nm emission).

## Data Availability

Not applicable.

## Sample Availability

Not available.

## References

[1] Kuetemeyer K., Lucas-Hahn A., Petersen B., Lemme E., Hassel P., Niemann H., Heisterkamp A. (2010). Combined multiphoton imaging and automated functional enucleation of porcine oocytes using femtosecond laser pulses. J. Biomed. Opt..

[2] Osychenko A., Zalessky A., Astafiev A., Shakhov A., Kostrov A., Krivokharchenko A., Nadtochenko V. (2020). Femtosecond laser-induced blastomere fusion results in embryo tetraploidy by common metaphase plate formation. Exp. Cell Res..

[3] Vogel A., Noack J., Hüttman G., Paltauf G. (2005). Mechanisms of femtosecond laser nanosurgery of cells and tissues. Appl. Phys. B.

[4] Babayan N., Vorobyeva N., Grigoryan B., Grekhova A., Pustovalova M., Rodneva S., Fedotov Y., Tsakanova G., Aroutiounian R., Osipov A. (2020). Low Repair Capacity of DNA Double-Strand Breaks Induced by Laser-Driven Ultrashort Electron Beams in Cancer Cells. Int. J. Mol. Sci..

[5] Wilson T.E., Sunder S. (2020). Double-strand breaks in motion: Implications for chromosomal rearrangement. Curr. Genet..

[6] Babayan N., Grigoryan B., Khondkaryan L., Tadevosyan G., Sarkisyan N., Grigoryan R., Apresyan L., Aroutiounian R., Vorobyeva N., Pustovalova M. (2019). Laser-Driven Ultrashort Pulsed Electron Beam Radiation at Doses of 0.5 and 1.0 Gy Induces Apoptosis in Human Fibroblasts. Int. J. Mol. Sci..

[7] Ulyanenko S., Pustovalova M., Koryakin S., Beketov E., Lychagin A., Ulyanenko L., Kaprin A., Grekhova A., Ozerova A.M., Ozerov I.V. (2019). Formation of gammaH2AX and pATM Foci in Human Mesenchymal Stem Cells Exposed to Low Dose-Rate Gamma-Radiation. Int. J. Mol. Sci..

[8] Osipov A.N., Pustovalova M., Grekhova A., Eremin P., Vorobyova N., Pulin A., Zhavoronkov A., Roumiantsev S., Klokov D.Y., Eremin I. (2015). Low doses of X-rays induce prolonged and ATM-independent persistence of γH2AX foci in human gingival mesenchymal stem cells. Oncotarget.

[9] Mehta A., Haber J.E. (2014). Sources of DNA double-strand breaks and models of recombinational DNA repair. Cold Spring Harb. Perspect. Biol..

[10] Abbotts R., Wilson D.M. (2017). Coordination of DNA single strand break repair. Free Radic. Biol. Med..

[11] Nuzzo V., Maxwell I., Chung S., Mazur E., Heisterkamp A. (2011). Subcellular Surgery and Nanoneurosurgery Using Femtosecond Laser Pulses. Biophotonics: Spectroscopy, Imaging, Sensing, and Manipulation.

[12] Osychenko A.A., Zalesskii A.D., Krivokharchenko A.S., Zhakhbazyan A.K., Ryabova A.V., Nadtochenko V.A. (2015). Fusion of blastomeres in mouse embryos under the action of femtosecond laser radiation. Efficiency of blastocyst formation and embryo development. Quantum Electron..

[13] Osychenko A.A., Tochilo U.A., Astafiev A.A., Zalessky A.D., Shakhov A.M., Krivokharchenko A.S., Nadtochenko V.A. (2017). Determining the Range of Noninvasive Near-Infrared Femtosecond Laser Pulses for Mammalian Oocyte Nanosurgery. Sovrem. Tehnol. Med..

[14] Botchway S.W., Reynolds P., Parker A.W., O’Neill P. (2012). Laser-Induced Radiation Microbeam Technology and Simultaneous Real-Time Fluorescence Imaging in Live Cells. Imaging and Spectroscopic Analysis of Living Cells-Optical and Spectroscopic Techniques.

[15] Harper J.V., Reynolds P., Leatherbarrow E.L., Botchway S.W., Parker A.W., O’Neill P. (2008). Induction of Persistent Double Strand Breaks Following Multiphoton Irradiation of Cycling and G1-arrested Mammalian Cells-Replication-induced Double Strand Breaks. Photochem. Photobiol..

[16] Ding D., Zhang Y., Wang J., Zhang X., Gao Y., Yin L., Li Q., Li J., Chen H. (2016). Induction and inhibition of the pan-nuclear gamma-H2AX response in resting human peripheral blood lymphocytes after X-ray irradiation. Cell Death Discov..

[17] Moeglin E., Desplancq D., Conic S., Oulad-Abdelghani M., Stoessel A., Chiper M., Vigneron M., Didier P., Tora L., Weiss E. (2019). Uniform Widespread Nuclear Phosphorylation of Histone H2AX Is an Indicator of Lethal DNA Replication Stress. Cancers.

[18] Venugopalan V., Guerra A., Nahen K., Vogel A. (2002). Role of Laser-Induced Plasma Formation in Pulsed Cellular Microsurgery and Micromanipulation. Phys. Rev. Lett..

[19] Vogel A., Venugopalan V. (2003). Mechanisms of Pulsed Laser Ablation of Biological Tissues. Chem. Rev..

[20] Hammer D.X., Thomas R.J., Noojin G.D., Rockwell B.A., Kennedy P.K., Roach W.P. (1996). Experimental investigation of ultrashort pulse laser-induced breakdown thresholds in aqueous media. IEEE J. Quantum Electron..

[21] Kudryashov S.I., Zvorykin V.D. (2008). Microscale nanosecond laser-induced optical breakdown in water. Phys. Rev. E.

[22] Oraevsky A.A., Da Silva L.B., Rubenchik A.M., Feit M.D., Glinsky M.E., Perry M.D., Mammini B.M., Small W., Stuart B.C. (1996). Plasma mediated ablation of biological tissues with nanosecond-to-femtosecond laser pulses: Relative role of linear and nonlinear absorption. IEEE J. Quantum Electron..

[23] Sarpe C., Köhler J., Winkler T., Wollenhaupt M., Baumert T. (2012). Real-time observation of transient electron density in water irradiated with tailored femtosecond laser pulses. New J. Phys..

[24] Stuart B.C., Feit M.D., Herman S., Rubenchik A.M., Shore B.W., Perry M.D. (1996). Nanosecond-to-femtosecond laser-induced breakdown in dielectrics. Phys. Rev. B.

[25] Perry C.F., Zhang P., Nunes F.B., Jordan I., von Conta A., Wörner H.J. (2020). Ionization Energy of Liquid Water Revisited. J. Phys. Chem. Lett..

[26] Fernando H., Papadantonakis G.A., Kim N.S., LeBreton P.R. (1998). Conduction-band-edge ionization thresholds of DNA components in aqueous solution. Proc. Natl. Acad. Sci. USA.

[27] Kennedy P.K. (1995). A first-order model for computation of laser-induced breakdown thresholds in ocular and aqueous media. I. Theory. IEEE J. Quantum Electron..

[28] Wang R., Zhang Q., Li D., Xu S., Cao P., Zhou Y., Cao W., Lu P. (2019). Identification of tunneling and multiphoton ionization in intermediate Keldysh parameter regime. Optics Express.

[29] Fan C.H., Sun J., Longtin J.P. (2002). Breakdown threshold and localized electron density in water induced by ultrashort laser pulses. J. Appl. Phys..

[30] Lenzner M., Krüger J., Sartania S., Cheng Z., Spielmann C., Mourou G., Kautek W., Krausz F. (1998). Femtosecond Optical Breakdown in Dielectrics. Phys. Rev. Lett..

[31] Boudaiffa B., Cloutier P., Hunting D., Huels M.A., Sanche L. (2000). Resonant formation of DNA strand breaks by low-energy (3 to 20 eV) electrons. Science.

[32] Gohlke S., Illenberger E. (2002). Probing biomolecules: Gas phase experiments and biological relevance. Europhys. News.

[33] Huels M.A., Boudaïffa B., Cloutier P., Hunting D., Sanche L. (2003). Single, Double, and Multiple Double Strand Breaks Induced in DNA by 3−100 eV Electrons. J. Am. Chem. Soc..

[34] Chin S.L., Hosseini S.A., Liu W., Luo Q., Théberge F., Aközbek N., Becker A., Kandidov V.P., Kosareva O.G., Schroeder H. (2005). The propagation of powerful femtosecond laser pulses in opticalmedia: Physics, applications, and new challenges. Can. J. Phys..

[35] König K. (2020). Review: Clinical in vivo multiphoton FLIM tomography. Methods Appl. Fluoresc..

[36] Müller D., Hagenah D., Biswanath S., Coffee M., Kampmann A., Zweigerdt R., Heisterkamp A., Kalies S.M.K. (2019). Femtosecond laser-based nanosurgery reveals the endogenous regeneration of single Z-discs including physiological consequences for cardiomyocytes. Sci. Rep..

[37] Chung S., Nuzzo V., Mazur E. Nanosurgery with Femtosecond Lasers. Proceedings of the Frontiers in Optics 2009/Laser Science XXV/Fall 2009 OSA Optics & Photonics Technical Digest.

